# Higher midlife CAIDE score is associated with increased brain atrophy in a cohort of cognitively healthy middle-aged individuals

**DOI:** 10.1007/s00415-020-10383-8

**Published:** 2021-01-09

**Authors:** Xulin Liu, Maria-Eleni Dounavi, Karen Ritchie, Katie Wells, Craig W. Ritchie, Li Su, Graciela Muniz-Terrera, John T. O’Brien

**Affiliations:** 1 Department of Psychiatry, School of Clinical Medicine, University of Cambridge, Level E4 Cambridge Biomedical Campus, Cambridge, CB2 0SP UK; 2 Centre for Dementia Prevention, University of Edinburgh Centre for Clinical Brain Sciences, Edinburgh, UK; 3 INSERM, University of Montpellier, Montpellier, France; 4 The Centre for Psychiatry, Imperial College London, London, UK

**Keywords:** Dementia, Alzheimer’s disease, Grey matter, Atrophy, Middle aged

## Abstract

**Background:**

Structural brain changes associated with Alzheimer’s disease (AD) can occur decades before the onset of symptoms. The Cardiovascular Risk Factors, Aging, and Dementia (CAIDE) score has been suggested to be associated with accelerated brain atrophy in middle-aged subjects but the regional specificity of atrophic areas remains to be elucidated.

**Methods:**

3T T1-weighted magnetic resonance imaging scans of 160 cognitively healthy middle-aged participants (mean age = 52) in the PREVENT-Dementia cohort, from baseline and from follow-up after 2 years, were examined. Images were preprocessed using Computational Anatomy Toolbox 12. Voxel-based morphometry was performed in FSL 6.0.1 to identify areas of grey matter (GM) volume differences both cross-sectionally and longitudinally between subjects with high and low baseline CAIDE score (CAIDE score was dichotomized at cohort-median). A GM percentage of change map was created for each subject for evaluation of atrophy over 2 years. Analyses were adjusted for age, gender, education and total intracranial volume.

**Results:**

Compared to subjects with CAIDE score ≤ 6 (low risk), subjects with CAIDE score > 6 (high risk) showed lower GM volume in the temporal, occipital, and fusiform cortex and lingual gyrus at baseline, and greater percentage of GM loss over 2 years in the supramarginal gyrus, angular gyrus, precuneus, lateral occipital cortex, superior parietal lobule and cingulate gyrus (corrected *P* < 0.05).

**Conclusion:**

This study demonstrated accelerated GM atrophy concentrated in several AD signature cortical regions in healthy middle-aged subjects with high CAIDE scores.

## Introduction

Dementia is characterized by cognitive decline, loss of memory and behavioral changes, leading to reduced ability to perform daily life activities [1]. By 2040, it is expected that the number of people with dementia will increase by 100% in developed countries and by approximately 300% in developing countries and heavily populated regions [2]. The most common cause of dementia is Alzheimer’s disease (AD) [3, 4]. Although AD symptoms usually appear after the age of 65, the potential neuropathology and brain structural alternations underlying AD might occur decades before the onset of symptoms [5–8]. Furthermore, exposure to many risk factors at middle age is associated with an increased risk of developing AD later in life [9]. Identification of AD structural biomarkers in middle-aged subjects at risk of future AD could, therefore, be an important step towards early diagnosis and interventions.

Cardiovascular risk factors are well-known risk factors for AD and are associated with brain volume changes commonly connected with AD [10–13]. Cross-sectional studies have found associations between cardiovascular risk factors (e.g., hypertension, obesity, high cholesterol) and decreases in total grey matter (GM) volume, hippocampal volume and cortical thickness [11–13]. The Cardiovascular Risk Factors, Aging and Dementia (CAIDE) score is a midlife dementia risk score calculated based on the population-based CAIDE study [14]. Some previous studies have demonstrated the association of CAIDE score with global brain volume and cognition. For example, a longitudinal study in cognitively healthy older people with a mean age of 70 years has shown that higher CAIDE scores were associated with lower total GM volume, lower cortical thickness, and worse cognition [11]. A cross-sectional study in healthy middle-aged subjects has demonstrated that higher CAIDE score was associated with poorer visual association learning and lower brain volume [15]. There has, however, been limited longitudinal research in cognitively healthy middle-aged people linking CAIDE score to longitudinal brain volume changes. A recently published longitudinal study in middle-aged subjects (aged 40–59) of the PREVENT-Dementia cohort has shown that having a midlife CAIDE score > 6 was associated with greater whole brain atrophy rate over 2 years [16]. However, the specific brain structures responsible for the increased brain atrophy with increased midlife CAIDE score are yet to be explored. This is important to determine as it might contribute to a better understanding of early brain structural atrophy underlying AD and provide specific targets for potential early interventions.

Hence, to address the current literature gap in the regional specificity of atrophic areas associated with dementia risk score in middle-aged people, the primary aims of this present study are to use voxel-based morphometry (VBM) to examine the specific brain regions with increased atrophy associated with higher CAIDE score and to examine the association of CAIDE score with cortical thickness.

## Materials and methods

### Study participants

This study examined participants of the PREVENT-Dementia cohort study (West London site), an ongoing multi-site longitudinal study in the UK and Ireland recruiting cognitively healthy volunteers in the age range of 40–59 [17]. Participants were recruited based on dementia family history, and their apolipoprotein E (APOE) genotype was recorded [17]. All subjects recruited in this cohort were cognitively unimpaired and details of baseline cognitive measures have been described elsewhere [15]. Magnetic resonance imaging (MRI) scans at baseline and at follow-up after 2 years were acquired. Participants who had completed both baseline and 2-year follow-up MRI scans by the time of this study were included (*n* = 168). One subject had missing essential information and, therefore, was excluded from image preprocessing. Hence, MRI scans of 167 subjects were included in image preprocessing.

### Dementia risk score

A CAIDE score was calculated for each participant in the PREVENT-Dementia cohort. The CAIDE score ranges from 0 to 18, calculated by taking into account age, gender, education years, systolic blood pressure, body mass index, total cholesterol, physical activity, and APOE ε4 carriership which is by far a major genetic risk factor for the development of sporadic AD [14, 18]. Midlife CAIDE score is highly predictive of dementia risk over 20 years and has been validated in a large (> 9000 participants) population from the US (age range, 40–55 years) [19]. In the present study, CAIDE score was treated as a binary variable dichotomized according to the group median (CAIDE score = 6) in accordance with the previous methodological approach in the research on the same cohort [16]. Baseline CAIDE score > 6 will be referred to as CAIDE-high and baseline CAIDE score ≤ 6 will be referred to as CAIDE-low in this study.

### MRI data acquisition and preprocessing

MRI scanning was carried out at the West London Cognitive Disorders Treatment and Research Unit in West London Mental Health Trust, West London, UK using a 3 T SIEMENS Magnetom Verio Syngo scanner. A three-dimensional T1-weighted magnetization prepared rapid gradient echo (MPRAGE) sequence image was acquired with repetition time = 2300 ms, echo time = 2.98 ms, FOV = 240 × 256 mm^2^, voxel size = 1.0 × 1.0 × 1.0 mm^3^, 160 slices with 1 mm slice thickness and flip angle = 9°.

The acquired T1-weighted MPRAGE images were preprocessed using the longitudinal processing stream of the Computational Anatomy Toolbox 12 (CAT12) (http://www.neuro.uni-jena.de/cat/) in Statistical Parametric Mapping 12 (https://www.fil.ion.ucl.ac.uk/spm/software/spm12/) based on MATLAB version 2019a (R2019a, MathWorks, Natick, MA, USA). As part of the longitudinal preprocessing, an initial intra-subject rigid registration was applied to realign images of all time points and an intra-subject bias correction was applied. Images were denoised and corrected for intensity non-uniformities and segmented into different tissue classes. Images were then spatially normalized using Diffeomorphic Anatomical Registration Through Exponentiated Lie Algebra (DARTEL) algorithm and registered to the Montreal Neurological Institute (MNI) 152 template. The resultant image in MNI space was modulated using the Jacobian determinants [20]. Finally, images were spatially smoothed with a 8 mm full-width at half maximum (FWHM) Gaussian kernel. The absolute and relative volumes of GM, white matter (WM), and cerebrospinal fluid (CSF) of the whole brain and the total intracranial volume (TIV) were calculated based on the segmented maps, as part of the CAT 12 preprocessing. Relative volume was calculated by dividing absolute volume by TIV, which would control for the individual differences in brain sizes. Cortical thickness was estimated as part of the preprocessing pipeline in CAT12 using the projection-based thickness approach [21].

All preprocessed GM images were inspected one-by-one visually for image quality assurance before further analysis. Images with poor quality or issues resulting from preprocessing that could not be addressed were excluded. Subjects with incidental findings such as meningiomas were excluded.

### Voxel-based morphometry

The smoothed, modulated, warped and segmented GM images generated from CAT12 preprocessing were used for voxel-wise [22] statistical analyses performed in FSL 6.0.1 (https://fsl.fmrib.ox.ac.uk/fsl/fslwiki/FSL). The GM images were investigated using voxel-wise general linear model (GLM) (https://fsl.fmrib.ox.ac.uk/fsl/fslwiki/GLM) with permutation nonparametric testing [23] (5000 permutations). Results were corrected for multiple comparisons in FSL by family-wise error (FWE) correction [24]. A FWE-corrected *P* value < 0.05 was considered to be statistically significant. Brain structures with significant difference between the considered groups were identified using the Harvard–Oxford Cortical and Subcortical Structural Atlases with an overlay of 20% or above of significant clusters with a particular structure. Baseline age, gender, education years and TIV were included as covariates in GLMs.

To limit the analysis to areas of GM, a GM mask was created by binarizing the individual GM images at a threshold of 0.1 (i.e., GM areas with GM > 10% were included) using MATLAB version 2019a (R2019a, MathWorks, Natick, MA, USA). For cross-sectional analyses to investigate any baseline difference, voxel-wise two-sample unpaired t-test was performed in FSL with a mask including all binarized individual baseline GM images to compare the GM volume between CAIDE-high and CAIDE-low subjects.

For longitudinal analyses, a percentage of change map was created for each subject as in the equation below:

$$100*\frac{{{\text{GM}}\,{\text{at}}\,{\text{followup}} - {\text{GM}}\,{\text{at}}\,{\text{baseline}}}}{{{\text{GM}}\,{\text{at}}\,{\text{baseline}}}}$$

where GM represents the 10% threshold-applied GM image (i.e., GM areas with GM > 10% were included). Non-numerical and infinite values were set to 0 in the change map. A mask for voxel-wise analysis was created by including all areas in the change maps with an absolute threshold of 0.01 (i.e., areas with absolute values > 0.01 were included) to exclude non-numerical and infinite values. Voxel-wise two-sample t-test was performed in FSL to compare the GM change maps between CAIDE-high and CAIDE-low subjects.

To further investigate the regions that showed significant differences in longitudinal GM volume change between the groups, an “AD signature cortical regions” mask was created by including the main cortical regions defined as “AD signature” in previous literature (inferior temporal gyrus, temporal pole, angular gyrus, superior frontal gyrus, superior parietal lobule, supramarginal gyrus, precuneus, inferior frontal sulcus) [25] using the Harvard-Oxford Cortical Structural Atlases in FSL. The voxels from regions showing significant differences in GM volume change between the groups that overlap with the AD signature cortical regions mask were quantified in FSL.

### Statistical analyses

Non-voxel-wise statistical analyses were performed in GraphPad Prism 8 (Version 8.3.1 (332)). Comparisons of demographic characteristics were performed using chi-squared tests for categorical variables and Mann–Whitney tests for continuous variables. The MRI measurements (i.e., relative volumes of GM, WM, and CSF; TIV; and cortical thickness) were compared cross-sectionally (i.e., at baseline) and longitudinally. The 2-year percentage of change of each measurement was calculated using the equation below:

$$100*\frac{{{\text{measurement}}\,{\text{at}}\,{\text{followup}} - {\text{measurement}}\,{\text{at}}\,{\text{baseline}}}}{{{\text{measurement}}\,{\text{at}}\,{\text{baseline}}}}$$

Linear regressions were used with MRI measurements as dependent variables in cross-sectional analyses and with the 2-year percentage of change of MRI measurements as dependent variables in longitudinal analyses. CAIDE score as a binary variable was used as an independent variable. As APOE ε4 carriership is included in the calculation of CAIDE score, post hoc analysis of APOE ε4 carriership was performed using it as an independent variable in linear regression to investigate whether the effect of CAIDE score was driven by APOE ε4 carriership. Age, gender and education years were included as covariates in regression analyses.

For all statistical analyses, demographic values and CAIDE score at baseline were used. *P* values < 0.05 were considered to be statistically significant.

## Results

### Characteristics of participants

Out of the 167 subjects with MRI scans preprocessed, 5 subjects were excluded due to incidental findings: 4 had meningioma and 1 had damage due to a brain surgery. Two subjects were excluded due to image quality issues in preprocessing. Hence, 160 was the final analytic sample. Baseline characteristics of participants are reported in Table 1. For those subjects with a family history of dementia, the mean age at which the first parent was diagnosed with dementia was 77 years, and therefore, these subjects were estimated at baseline to be approximately 24 years away from possible dementia onset based on their parental age at diagnosis.

**Table 1:** Baseline characteristics of study participants

	All subjects	CAIDE score > 6	CAIDE score ≤ 6	*P* value
*n*	160	68	92	
CAIDE score	5.9 ± 2.9	8.5 ± 1.6	4.0 ± 2.0	
Age	52.0 ± 5.3	55.0 ± 3.7	49.8 ± 5.3	< 0.001
Female *n* (%)	114 (71.2)	42 (61.8)	72 (78.3)	0.023
Education years	16.1 ± 3.4	15.3 ± 3.4	16.6 ± 3.3	0.028
APOE ε4 carriers *n* (%)	61 (38.1)	39 (57.4)	22 (23.9)	< 0.001
SBP (mmHg)	121.6 ± 14.7	127.1 ± 15.3	117.5 ± 12.8	< 0.001
BMI (kg/m^2^)	27.1 ± 4.7	29.2 ± 5.3	25.6 ± 3.4	< 0.001
Total cholesterol (mmol/L)	5.6 ± 1.0	5.7 ± 1.2	5.5 ± 0.9	0.25
Physical activity (*n* and % of subjects defined as active)	33 (20.6)	11 (16.2)	22 (23.9)	0.23
FHD + *n* (%)	86 (53.8)	43 (63.2)	43 (46.7)	0.039

Plus-minus values are means ± standard deviation. *P* value represents the *P* value of statistical difference between subjects with CAIDE score > 6 and CAIDE score ≤ 6

*APOE* apolipoprotein E, *BMI* body mass index, *CAIDE* Cardiovascular Risk Factors, Aging, and Dementia risk, *FHD* + with family history of dementia, *SBP* systolic blood pressure

### Analyses of whole brain tissues

For the whole sample on average, from baseline to follow-up, GM volume decreased by 0.96% ± 1.9%, cortical thickness decreased by 0.5% ± 1.0%, WM decreased by 0.12% ± 1.7%, and CSF increased by 2.2% ± 5.3%.

Cross-sectionally, CAIDE score was negatively associated with GM volume and positively associated with CSF volume (Table 2). Longitudinally, CAIDE score was associated with decreases in GM volume and cortical thickness and with an increase in CSF volume (Table 2), as expected from previously published analyses of this cohort [16]. After adjusting for age, gender and education years, higher CAIDE score remained associated with a decrease in GM volume (Table 2). APOE ε4 allele carriership was not a significant predictive variable of any tissue volume or cortical thickness cross-sectionally or longitudinally.

**Table 2:** Results of the linear regressions with brain volumes and cortical thickness at baseline and the percentage of change^a^ over 2 years as dependent variables and with binary CAIDE score as an independent variable

	CAIDE score univariate	CAIDE score covariate^b^
GM^c^ (%)		
Baseline	β = − 0.098, SE = 0.030, *P* = 0.0016*	β = − 0.54, SE = 0.36, *P* = 0.14
Percentage of change	β = − 0.065, SE = 0.021, *P* = 0.0020*	β = − 0.0077, SE = 0.0034, *P* = 0.026*
Cortical thickness (mm)		
Baseline	β = − 0.045, SE = 0.029, *P* = 0.13	β = − 0.0053, SE = 0.034, *P* = 0.87
Percentage of change	β = − 0.061, SE = 0.024, *P* = 0.014*	β = − 0.053, SE = 0.029, *P* = 0.064
CSF^c^ (%)		
Baseline	β = 0.064, SE = 0.031, *P* = 0.042*	β = 0.020, SE = 0.036, *P* = 0.57
Percentage of change	β = 0.071, SE = 0.026, *P* = 0.0065*	β = 0.055, SE = 0.030, *P* = 0.067
WM^c^ (%)		
Baseline	β = − 0.0025, SE = 0.028, *P* = 0.93	β = 0.016, SE = 0.032, *P* = 0.62
Percentage of change	β = − 0.033, SE = 0.021, *P* = 0.13	β = − 0.019, SE = 0.025, *P* = 0.45
TIV (cm^3^)		
Baseline	β = 0.055, SE = 0.029, *P* = 0.059	β = 0.022, SE = 0.027, *P* = 0.42
Percentage of change	β = 0.012, SE = 0.017, *P* = 0.48	β = 0.012, SE = 0.020, *P* = 0.53

*CAIDE* Cardiovascular Risk Factors, Aging, and Dementia risk, *CSF* cerebrospinal fluid, *GM* grey matter, *SE* standard error, *TIV* total intracranial volume, *WM* white matter

^a^Percentage of change over 2 years was calculated by (measurement at follow-up – measurement at baseline)/measurement at baseline

^b^CAIDE score was used as an independent variable with age, gender, and education years added as covariates in linear regressions

^c^Relative volume was used in analyses, calculated by absolute volume/TIV and represented as a percentage

^*^Statistically significance defined as *P* < 0.05

### Voxel-based morphometry

**Fig. 1:**
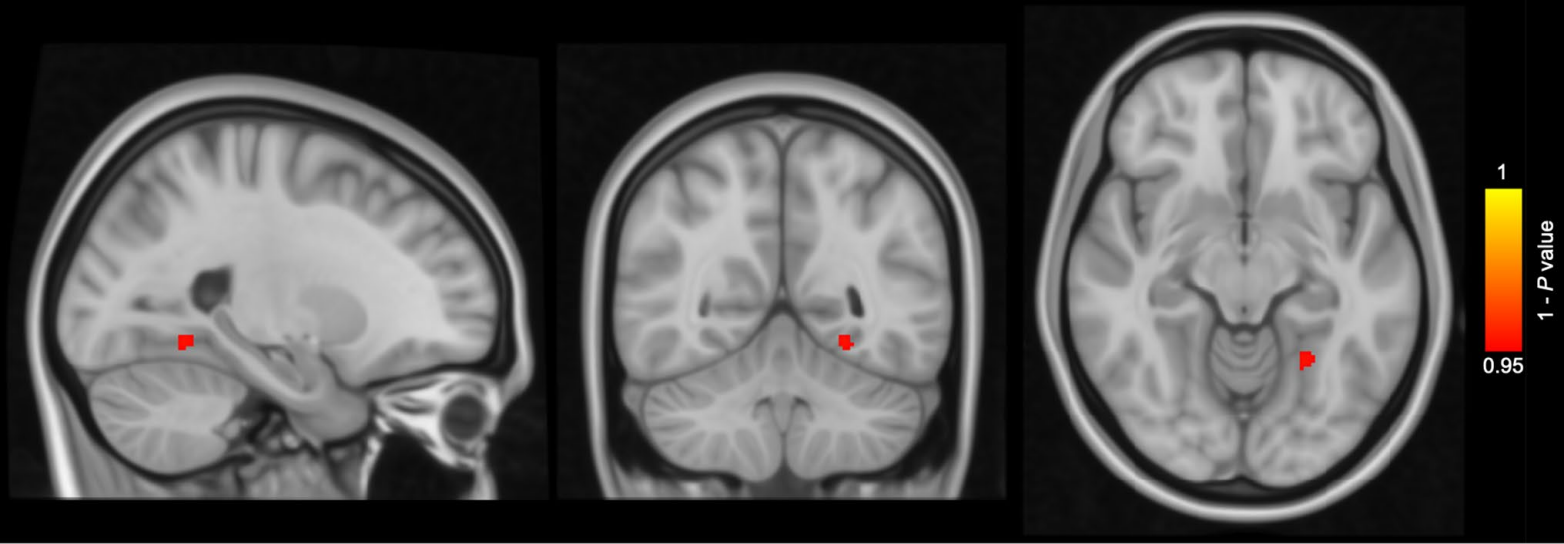
Voxel-based morphometry results of cross-sectional grey matter (GM) comparison between subjects with Cardiovascular Risk Factors, Aging, and Dementia risk (CAIDE) score > 6 (*n* = 68) and subjects with CAIDE score ≤ 6 (*n* = 92) at baseline. Areas in red-yellow represent areas, where the GM volume was lower in subjects with CAIDE score > 6 than subjects with CAIDE score ≤ 6 (family-wise error corrected *P* < 0.05), adjusting for age, gender, education years and total intracranial volume

**Fig. 2:**
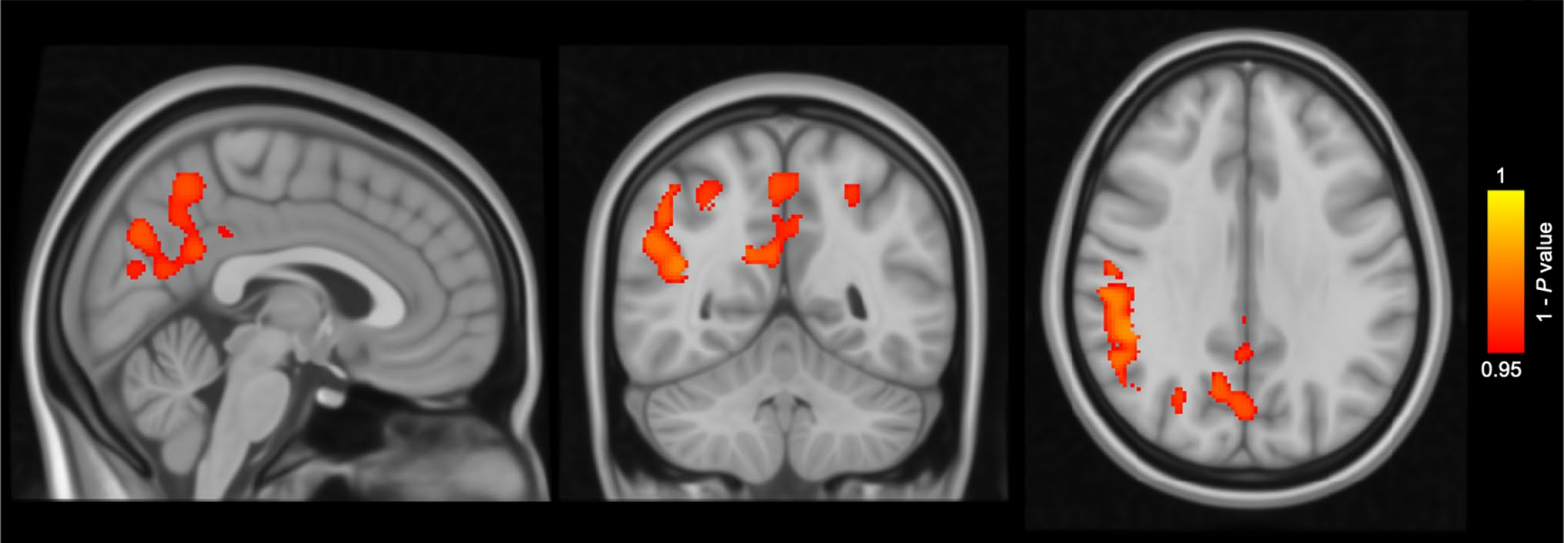
Voxel-based morphometry results of comparison of the grey matter (GM) percentage of change over 2 years between subjects with Cardiovascular Risk Factors, Aging, and Dementia risk (CAIDE) score > 6 (*n* = 68) and subjects with CAIDE score ≤ 6 (*n* = 92). Areas in red-yellow represent areas, where the percentage of GM loss was greater in subjects with CAIDE score > 6 than subjects with CAIDE score ≤ 6 (family-wise error corrected *P* < 0.05), adjusting for age, gender, education years and total intracranial volume

Cross-sectionally, CAIDE-high subjects showed smaller GM volume in the temporal, occipital, and fusiform cortex and lingual gyrus in comparison with CAIDE-low subjects at baseline (Fig. 1). Longitudinally, CAIDE-high subjects showed greater percentage of GM volume loss compared to CAIDE-low subjects in the supramarginal gyrus, angular gyrus, precuneus, lateral occipital cortex, superior parietal lobule and cingulate gyrus (Fig. 2). VBM results with cluster details are shown in Table 3. 76.6% of the regions showing greater percentage of GM volume loss in CAIDE-high subjects were located within the AD signature cortical regions [25] (Table 4).

**Table 3:** VBM results of GM volume comparison between subjects with CAIDE score > 6 and subjects with CAIDE score ≤ 6

Anatomical region	Cluster size (voxels)	Coordinates (MNI)			FWE-corrected *P* value
		*x*	*y*	*z*	
*Cross-sectional*					
Temporal, occipital and fusiform cortex; Lingual gyrus	39	− 24	− 54	− 10.5	0.047
*Longitudinal*					
Supramarginal gyrus; Angular gyrus	4109	51	− 42	34.5	0.020
Precuneus	2270	15	− 57	25.5	0.031
Lateral occipital cortex	342	43.5	− 79.5	− 1.5	0.038
Superior parietal lobule	92	− 25.5	− 54	49.5	0.039
Cingulate gyrus	46	3	− 37.5	33	0.049

*CAIDE* Cardiovascular Risk Factors, Aging, and Dementia risk, *FWE* family-wise error, *GM* grey matter, *MNI* Montreal Neurological Institute, *VBM* voxel-based morphometry

**Table 4:** Cluster sizes of AD signature cortical regions^a^, regions showing increased GM atrophy in subjects with CAIDE score > 6, and their overlapped regions

Anatomical region	Cluster size (voxels)
AD signature cortical regions	101,020
Regions with increased GM atrophy in subjects with CAIDE score > 6	6859
AD signature cortical regions with increased GM atrophy in subjects with CAIDE score > 6	5255

*AD* Alzheimer’s disease, *CAIDE* Cardiovascular Risk Factors, Aging, and Dementia risk, *GM* grey matter

^a^AD signature cortical regions include eight main regions (inferior temporal gyrus, temporal pole, angular gyrus, superior frontal gyrus, superior parietal lobule, supramarginal gyrus, precuneus, inferior frontal sulcus)[25]

## Discussion

Following a recent study reporting the association of CAIDE score with global GM atrophy and ventricular enlargement in the PREVENT-Dementia cohort [16], the present study has investigated GM associations with CAIDE score at a voxel-wise level to identify specific structures with accelerated GM atrophy. The present study has also investigated the association of CAIDE score with cortical thickness. Our results for the global tissue volumes are in line with the previous study [16], using in the present study a different image processing software tool. Using VBM the current study has investigated the voxel-wise associations between GM volume and CAIDE score and has localized specific regions with increased GM loss predicted by a baseline CAIDE score greater than 6. We have shown that in cognitively healthy middle-aged subjects a CAIDE score greater than 6 was associated with lower GM volume in the temporal, occipital, and fusiform cortex and lingual gyrus cross-sectionally and with accelerated GM atrophy longitudinally in the supramarginal gyrus, angular gyrus, precuneus, lateral occipital cortex, superior parietal lobule and cingulate gyrus. These results were adjusted for the effects of age, gender, education and TIV.

The structures demonstrating accelerated atrophy with a high CAIDE score are involved in the process of consciousness, integration of elements, visual-spatial memory, and episodic memory according to the literature [26–29]. The majority of these regions could be classified as “AD signature cortical regions”, where AD-related cortical thinning often occurs [25] and such thinning has been suggested to be related to greater likelihood of progression from mild cognitive impairment to mild AD [30] and related to symptom severity in mild AD [25]. Based on findings from the present study, some of these AD signature cortical regions are vulnerable to accelerated GM atrophy associated with a high CAIDE score in midlife. In previous studies, CAIDE score has been more commonly examined as a continuous variable [11, 15, 31, 32]. A longitudinal study on a Finnish population has shown that increased midlife CAIDE score was associated with lower total GM volume and lower AD signature cortical thickness [11]. We have also examined the association between CAIDE score and cortical thickness in this present study and found that a CAIDE score > 6 was associated with increased cortical thinning although the association did not survive after adding age, gender and education years as covariates. The Finnish study has also found that higher midlife CAIDE score was associated with lower hippocampal volume, more pronounced deep WM lesions, more pronounced visually rated medial temporal atrophy (MTA) and poorer cognition later in life [11]. Higher midlife CAIDE score has been found to be associated with more severe MTA up to 30 years later also in another Finnish study [31] and in a cross-sectional study of non-demented subjects in Sweden [32]. We have found in this present study that higher CAIDE score was associated with accelerated GM atrophy in approximately 5% of the AD signature cortical regions [25] but not the medial temporal lobe. The possible reason for the discrepancy might be that the previous longitudinal studies mentioned above followed subjects for an average of more than 15 years, while this present study was based on longitudinal analyses over only 2 years. In the long run MTA might be associated with high midlife CAIDE score but short-term changes associated with high CAIDE score in midlife might be mostly concentrated in regions shown in this present study. Increased atrophy in these regions might, therefore, suggest an increased vulnerability to AD indicated by higher CAIDE score during midlife. The present results also suggest that CAIDE score might be a sensitive score to assist in detecting potentially AD-related brain structural alternations in cognitively healthy middle-aged subjects.

CAIDE score was calculated based on age, gender and education in addition to vascular risk factors and APOE genotype. Since age has been suggested to be the largest contributor to the dementia risk predictive effect of CAIDE score [33], this present study, in addition to analyzing CAIDE score as a single independent variable, also analyzed CAIDE score with age, gender and education years as covariates to control for any linear effects of these factors. The voxel-wise association between CAIDE score and GM atrophy remained significant after controlling for the effects of age, gender, and education years. In addition, APOE genotype in this cohort was not associated with GM volume loss over time. Hence, the association between CAIDE score and GM atrophy observed in VBM analyses might be mostly driven by vascular factors within the CAIDE score. This is of particular importance considering that around a third of AD cases worldwide might be attributable to potentially modifiable risk factors, including some risk factors that are taken into account when calculating the CAIDE score, such as midlife hypertension, midlife obesity, physical inactivity, and low education [10]. The increased GM loss in middle-aged subjects with a higher dementia risk score suggests that the observed accelerated GM atrophy in several AD-signature regions might potentially be delayed by improving modifiable vascular risk factors.

Although baseline volume differences were found between CAIDE-high and CAIDE-low subjects in a very small number of areas, these areas did not show differential degree of atrophy over time between the groups. This possibly suggests that the baseline differences found in this study had been established in the previous years of life and the rate of atrophy in these areas during midlife might not be significantly associated with the risk of dementia as defined by CAIDE score. Given that only a very small number of areas showed subtle baseline differences, the differences could also be attributable to inherited volume differences between individuals, and therefore, it is reasonable that some regions showing baseline differences are generally unaffected in AD. On the other hand, it is important to indicate that the cortical regions with differences in longitudinal GM atrophy between groups did not show any difference at baseline. One possible explanation is that these subjects might be at the age when AD signature cortical regions are just beginning to show accelerated atrophy associated with a higher risk of dementia as predicted by CAIDE score, suggesting that possible interventions should be implemented at this age or even earlier.

Strengths of this study include its longitudinal design. The longitudinal image preprocessing stream in CAT12 used in this study was developed and optimized to detect more subtle effects over shorter periods in comparison with traditional cross-sectional preprocessing pipelines and, therefore, conferred an advantage in this middle-aged and cognitively healthy population, in which any differences related to dementia risk might be more subtle than those normally observed in older age over longer periods of time. CAT12 pipeline is also considered an advanced but computationally less expensive tool in brain volume segmentation [34]. A second strength is that CAIDE score was originally developed in middle-aged subjects, and therefore, it is the most appropriate and sensitive for use in assessments of middle-aged people. Finally, age, gender and education years were adjusted in VBM analyses, and therefore, the increased atrophy associated with high CAIDE score should mainly indicate the impact of vascular factors.

A limitation of this study is that the actual future incidence of dementia in these subjects remains unknown and requires long-term follow-up to reveal the association between CAIDE score, brain atrophy and cognitive decline to a dementia syndrome. Additionally, it should be noted that subjects in the PREVENT-Dementia cohort were all volunteering participants. They might not perfectly reflect the general population, as they were likely to be more concerned about their general health and have been more proactively reducing modifiable risk factors for dementia.

## Conclusion

To our knowledge, this is the first longitudinal study to demonstrate accelerated GM atrophy concentrated in the supramarginal gyrus, angular gyrus, precuneus, lateral occipital cortex, superior parietal lobule and cingulate gyrus at middle age associated with high CAIDE score. These findings highlight the possibility and significance of early AD intervention through modifiable midlife vascular risk factors. AD diagnosis and interventions should be implemented early before symptoms occur. Interventions could, in light of present findings of subtle longitudinal GM volume changes occurring in midlife, possibly be informed by CAIDE score and implemented during or even earlier than middle age. Planned longitudinal analysis of the whole PREVENT-Dementia cohort (target recruitment: 700 people) to confirm the specific structures of accelerated atrophy associated with high CAIDE score in midlife and long-term follow-up to investigate the impact on clinical cognitive performance would be valuable. Further investigation into the specific midlife vascular risk factors accelerating GM loss in these regions would be important for the potential development of disease prevention and early intervention strategies.

## Data Availability

Data are available upon reasonable request.

## Code availability

Available upon reasonable request.
